# Bioprospecting of endophytic fungi from medicinal plant *Anisomeles indica* L. for their diverse role in agricultural and industrial sectors

**DOI:** 10.1038/s41598-023-51057-5

**Published:** 2024-01-05

**Authors:** Prabha Toppo, Pooja Jangir, Namita Mehra, Rupam Kapoor, Piyush Mathur

**Affiliations:** 1 Microbiology Laboratory, Department of Botany, University of North Bengal, Rajarammohunpur, Dist. Darjeeling, West Bengal 734013 India; 2 Plant-Fungus Interactions Laboratory, Department of Botany, University of Delhi, Delhi, 110007 India

**Keywords:** Fungi, Industrial microbiology, Plant sciences, Plant symbiosis

## Abstract

Endophytes are microorganisms that inhabit various plant parts and cause no damage to the host plants. During the last few years, a number of novel endophytic fungi have been isolated and identified from medicinal plants and were found to be utilized as bio-stimulants and bio fertilizers. *In lieu of this*, the present study aims to isolate and identify endophytic fungi associated with the leaves of *Anisomeles indica* L. an important medicinal plant of the Terai-Duars region of West Bengal. A total of ten endophytic fungi were isolated from the leaves of *A. indica* and five were identified using ITS1/ITS4 sequencing based on their ability for plant growth promotion, secondary metabolite production, and extracellular enzyme production. Endophytic fungal isolates were identified as *Colletotrichum yulongense* Ai1, *Colletotrichum cobbittiense* Ai2, *Colletotrichum alienum* Ai2.1, *Colletotrichum cobbittiense* Ai3, and *Fusarium equiseti*. Five isolates tested positive for their plant growth promotion potential, while isolates Ai4. Ai1, Ai2, and Ai2.1 showed significant production of secondary metabolites viz. alkaloids, phenolics, flavonoids, saponins, etc. Isolate Ai2 showed maximum total phenolic concentration (25.98 mg g^−1^), while isolate Ai4 showed maximum total flavonoid concentration (20.10 mg g^−1^). Significant results were observed for the production of extracellular enzymes such as cellulases, amylases, laccases, lipases, etc. The isolates significantly influenced the seed germination percentage of tomato seedlings and augmented their growth and development under in vitro assay. The present work comprehensively tested these isolates and ascertained their huge application for the commercial utilization of these isolates both in the agricultural and industrial sectors.

## Introduction

Endophytic fungi invariably inhabit all plant parts and do not cause any harmful effects on the host plants^1^. A number of studies have reported the isolation of large groups of endophytic fungi from the leaves of medicinal plants that have notable importance^2–7^. These endophytic fungi associated with medicinal plants have shown to possess the ability to produce new leads of secondary metabolites such as alkaloids, flavonoids, terpenoids, lignans, coumarins, glycosides, quinones as well as steroids^8–12^. Apart from the above, these endophytes also stimulate plant growth and development along with the secretion of various enzymes that have industrial and biotechnological implications^13,14^.

Secondary metabolites from medicinal plants play a crucial role as most of these metabolites have bioactive properties that can be further exploited in the pharmaceutical industry^15^. There are reports that state the production of these bioactive secondary metabolites in medicinal plants is stimulated by endophytic fungi, and close association of these endophytic fungi will lead to augmentation of these secondary metabolites in host plants^16–20^. Interestingly, the isolation of these endophytic fungi associated with medicinal plants will be highly beneficial as this will help in the large-scale production of bioactive secondary metabolites with medicinal value^21^. Moreover, studies on endophytes have also delineated that these endophytes can produce distinctive metabolites, and sometimes these metabolites act as precursors for the production of secondary compounds in distinct pathways; for example, plumbagin^22^, Huperzine A^23^, the anti-cancerous drug Rhytidchromones A^24^, etc. Previous studies have reported the isolation of novel compounds from these endophytic fungi that have been used for the treatment of various ailments^25–29^. A study showed that endophytic fungi *Penicillium canescens*, *P. murcianum*, *Paraphoma radicina*, and *Coniolariella hispanica* are independent producers of cryptotanshinone (diterpenoid compound), which is the main component of metabolite as the host *Salvia abrotanoides* plant^30^. Likewise, some studies stressed upon the production of important bioactive secondary metabolites from endophytic fungi like mellein and β-retinaldehyde from *Botryosphaeria fabicerciana*^31^; vinblastine from *Fusarium solani* RN1 and *Chaetomium funicola* RN3^32^; 3-hydroxy-4-(hydroxy(4-hydroxyphenyl) methyl) dihydrofuran-2-on from endophytic fungus *Fusarium verticillioides*^33^. These diverse types of fungal metabolites have several unique properties like anti-oxidant, anti-microbial, anti-malarial, anti-cancerous, anti-respiratory syncytial, and cytotoxic activities^34,35^. For instance, an endophytic fungus *Curvularia papendorfii*, produced the bioactive compound mannitol and kheiric acid, which have anti-viral, anti-bacterial as well as anti-proliferative activity against pathogens such as the human coronavirus HCoV 229E, the feline coronavirus FCV F9 and pathogenic bacteria like *Staphylococcus* sp.^36^. Similarly, another study reported production of a unique fungal metabolite like anofinic acid with acute anti-microbial activity against both gram-positive and gram-negative bacteria such as *Pseudomonas aeruginosa*, *Staphylococcus aureus, Escherichia coli, and Bacillus subtilis* from endophytic fungus *Aspergillus tubingensis* ASH4^37^. Widjajanti and co-workers^38^, isolated the fungi *Phyllosticta* sp. from *Hippobroma longiflora* and reported production of metabolites like alkaloids, flavonoids, and terpenoids along with high anti-oxidant activity in ethyl acetate extracts. A recent review by Toppo et al.^39^ depicted the production of several secondary metabolites by endophytic fungi and their utilization in different industries.

Endophytic fungi have been shown to escalate plant growth through phosphate solubilization and the production of indole-3-acetic acid (IAA) and ammonia^40,41^ (NH_3_). Endophytic fungi *Alternaria* sp., *Didymella* sp., *Fusarium* sp., and *Xylogone* sp. isolated from *Sophora flavescens* reported for IAA production and phosphate solubilization activities^42^. Similarly, Roy et al.^43^, reported that the endophytic fungi *Colletotrichum* sp. associated with the *Plumbago zeylanica*, a medicinal plant had a considerable impact on plant growth parameters as evident from positive results of phosphate solubilisation activity, ammonia, and IAA production under in vitro conditions. Soni et al.^44^, also reported that endophytic fungi derived from *Bacopa monnieri* plant have substantial potential to solubilize phosphate and produce IAA.

A number of extracellular hydrolytic enzymes, such as cellulases, amylases, lipases, etc., have been observed to be secreted by these endophytic fungi under in vitro conditions and utilization of these endophytic fungi strains will largely help in bioremediation and industrial purposes^45–49^. Jagannath and co-worker^14^, reported significant production of extracellular enzymes such as amylases, cellulases, phosphates, proteases, and lipases from varied classes of endophytic fungi associated with a medicinal plant *Baliospermum montanum.* Concomitant reports were observed in the work of Sopalun and Iamtham^7^, that exhibited production of different extracellular enzymes such as pectinase, amylase, protease, cellulase, and lipase by different endophytic fungi isolated from roots, stem, and leaves of different Thai orchids. Matias et al.^50^, observed that the endophytic fungi, *Aspergillus* sp. A9, *Aspergillus* sp. A36, *Penicillium* sp. P5, and *Penicillium* sp. P15 derived from medicinal plants *Myrcia guianensis,* was immensely producing amylases, lipases, proteases, and xylanases. The study also showed that these strains can have been used for the removal of *Staphylococcus aureus* biofilms. Jagannath et al.^14^, reported that twenty-nine endophytic fungi isolated from *Baliospermum montanum*, a medicinal plant distributed throughout India, had the capability to produce amylase (83% of isolates), cellulase (79% of isolates), phosphatase (77% of isolates) protease (72% of isolates), and lipase (59% of isolates). A number of these extracellular enzymes are being used in textile, pharmaceutical, and also in agricultural sectors^7^. Furthermore, these fungal strains are of utmost importance for their high production capability, easy availability, a higher growth rate, which assist in enzyme production that can be used in several fields, including biodegradation of dead organic material, food processing, pharmaceuticals, and also for agricultural purposes, etc.^51^.

*Anisomeles indica* L., popularly known as Indian Catmint, belonging to the family Lamiaceae is a specific perennial woody shrub, with a broad range of medicinal properties. It is an aromatic plant that grows in the wild and is found in various parts of Southeast Asia including some parts of India^52,53^. Terai-Duars is situated in the northernmost part of the state of West Bengal, India, along the foothills of the North-Eastern Himalayas and is known for its richest biodiversity with ideal climatic environments that favours and adds richness to the medicinal value of *A. indica* plants^54^. Extracts of *A. indica* plant contain different biochemical active constituents like diterpenoids, benzenoids, flavonoids, and phenylpropanoids^55–57^ and have been shown to possess a phytotoxic effect^58–60^. The plant leaf extract is also known for its anti-inflammatory, anti-bacterial, and anti-oxidative effects on a number of diseases^61,62^. Although there are studies carried out to study the medicinal properties of this plant, none of the studies have been carried out to investigate the diversity of endophytic fungi associated with this plant.

Therefore, the present study aims to isolate endophytic fungi from *A. indica* and bioprospection of these endophytic fungi for secondary metabolites, plant growth promotion, and enzymes of commercial importance. The work will be the first report regarding the study of endophytic diversity associated with a medicinal plant of this region and will delineate the possible functional role of these beneficial microbes.

## Materials and methods

### Isolation of endophytic fungi

**Figure 1:**
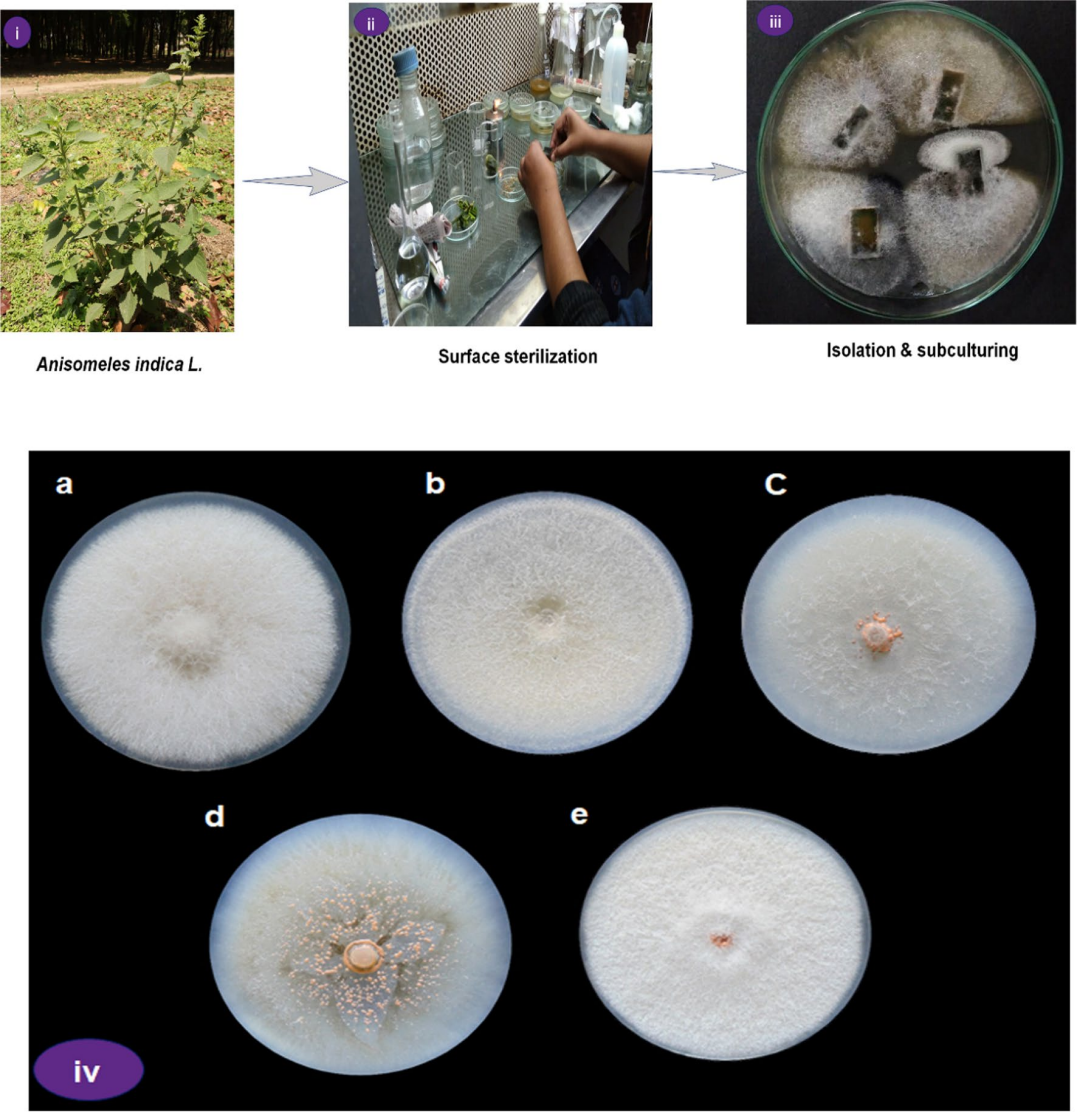
Schematic presentation of the procedure of isolation of endophytic fungal isolates from the leaves of *A. indica*. (**a**) Healthy plant of *A. indica* was collected from the campus of University of North Bengal (**i**); Leaf segments were cut into small pieces and surface sterilized under aseptic conditions (**ii**); Sterilized leaf segments were placed onto the fresh potato dextrose agar (PDA) plates for the isolation of endophytes (**iii**). (**b**) Five distinct endophytic fungal isolates were screened growing on PDA plates for morphological differences.

Plants of *Anisomeles indica* L. were collected from the campus of the University of North Bengal (Fig. 1i). Experimental research and field studies on the plants (either cultivated or wild), including the collection of the plant material are in compliance with relevant institutional, national, and international guidelines and legislation.

Isolation of endophytic fungi was carried out from mature, symptomless, and healthy surface-sterilized leaves following the method of Teimoori-Boghsani et al.^30^, with slight modifications. The leaves were cut into small segments and immersed in 70% ethanol for 1 min, followed by 2.5% sodium hypochlorite treatment for 3 min; further segments were washed using sterilized distilled water and finally immersed in 70% ethanol for the 30 s (Fig. 1ii). The sterilized segments were cut aseptically into small segments (Fig. 1iii) and were placed very carefully on potato dextrose agar (PDA) plates augmented with 100 mg mL^−1^ streptomycin to avoid bacterial contamination. Plates were incubated at 25 ± 2 °C for 5–7 days until the fungal colonies emerged from leaf sections. The growing hyphal tips of fungal colonies were transferred onto fresh PDA plates until the pure cultures were obtained.

### DNA isolation and molecular identification

All the fungal isolates were assessed based on their morphological and microscopic features for preliminary identification. Fungal isolates were grown on potato dextrose broth (PDB) for 7 days to get a fungal mycelial mat to isolate DNA. One gram of mycelial mat was harvested and ground to a fine powder using liquid nitrogen using a sterilized pestle and mortar. The CTAB-Chloroform method was used to isolate fungal DNA using an extraction buffer (100 mM Tris HCL, 20 mM EDTA, 2% CTAB, 1.4 M NaCl). Further, molecular identification was carried out using ITS-rDNA sequencing followed by the protocol with slight modifications^63^. The fungal internal transcribed spacer (ITS) regions were amplified with the primers of ITS-1 (5-TCCGTAGGTGAACCTGCGG-3) and ITS-4 (5-TCCTCCGCTTATTGATATGC-3)^64^. PCR reaction mixtures contained 25 mM MgCl_2_, PCR buffer, 10 mM dNTP mix, 10 μM of each primer ITS1 and ITS4, 5 U μL^−1^ Taq DNA polymerase (Promega, Madison, WI, USA), and nearly 25 ng μL^−1^ of the extracted genomic DNA. PCR was performed in a DNA engine thermal cycler (Eppendorf, Mastercycler^@^ nexus gradient) with 94 °C for 4 min, followed by 25 cycles of 94 °C for 1 min, 55 °C for 1 min, and 72 °C for 2 min, followed by final extension performed at 72 °C for 10 min. The PCR products were checked on 1.2% agarose gel electrophoresis in 1 × TAE Buffer (Tris–acetate–EDTA). The PCR products were commercially sequenced by Barcode Biosciences, India. The amplified PCR products were checked for the probable size on 1.2% agarose gel and the final product was commercially sequenced by Barcode Biosciences, India. The ITS sequences of the fungal isolates were deposited in NCBI GenBank for accession numbers. The phylogenetic tree was constructed using the MEGA X software by comparing similar sequences from the NCBI database by the neighbor-joining method with 1000 repeats bootstrap analysis.

### Plant growth promoting traits

#### Phosphate solubilization activity

For the determination of phosphate solubilization activity, the fungal strains were cultured in freshly prepared PDA plates and incubated for 7 days at 25 ± 2 °C. After that, the fungal stubs were inoculated on Pikovskaya’s agar plate (Himedia, India) containing Ca_3_(PO_4_)_2_ with the help of a cork borer and kept in an incubator for 5–6 days. The appearance of the clear halo zone around the inoculated spot was observed as positive for phosphatase production. The diameter of clear zones around the fungal colony indicated the phosphate solubilizing potential of the isolates^65^.

#### NH_3_ production

NH_3_ production ability was studied by inoculating endophytic fungal isolates into the peptone water (peptone 10 g; NaCl 5 g; in 1 L of distilled water) media and incubated for 5 days in the dark on a rotary shaker with 120 rpm at 25 ± 2 °C. Following incubation, 0.2 mL culture supernatant was mixed with 1 mL of Nessler’s reagent. The appearance of a yellowish-brown color demonstrated NH_3_ production by the isolates^41^.

#### IAA production

Estimation of IAA production was done following the spectrophotometric method of Yadav et al.^66^, with minor modifications. The endophytic fungi were grown in Luria broth (LB) supplemented with L-tryptophan (2 mg/mL) for 6 days at 25 ± 2 °C. Non-inoculated media was used as a control. After incubation, each fungal culture broth was centrifuged at 10,000 rpm for 10 min. 1 mL of supernatant was allowed to react with 2 mL of Salkowski reagent (HiMedia). The appearance of pink colour signified the presence of IAA, and the absorbance was measured at 530 nm. The concentration of IAA was estimated by preparing a standard curve from commercially available IAA (Sigma, India) and the quantity of IAA was expressed as µg mL^−1^.

#### Preparation of fungal crude extracts and the separation of bioactive compounds

The actively growing fungal mycelial plugs were cut into 5 mm diameters and inoculated into 200 mL of PDB in 500-mL Erlenmeyer flasks. The flasks were incubated in dark for 15–21 days at 25 ± 2 °C in a rotary shaker incubator at 120 rpm^67^. The fungal mat was separated through sterilized muslin cloth and the broth was centrifuged to separate any leftover mycelial residue from the broth. The filtrate was extracted by using an equal volume of ethyl acetate (1:1, v/v) in a 250 mL separating funnel. Then, the organic layer of ethyl acetate (EAE) containing bioactive compounds was separated thrice and evaporated using a hot air oven at 35–40 °C for 24 h. Further, the mass of mycelium was dried at 40 °C for 24 h in a hot-air oven and ground into a fine powder with a mortar and pestle using ethyl acetate. Both the crude extracts were collected, and the residue was stored at 4 °C for further analysis.

#### Screening for secondary metabolites

Crude extracts were qualitatively assessed for the presence of various classes of active chemical constituents according to standard protocols followed for alkaloids^68^, flavonoids^69^, saponins^68^, phenols^70^, steroids^70^, and terpenoids^70^.

#### Quantitative estimation for total phenols and flavonoids

The crude extracts of all the fungal isolates were prepared using the protocol of Gunasekaran et al.^71^, and the content of total phenols was estimated using the Folin-Ciocalteu method. A sample of crude fungal extract of 10 mg mL^−1^ was mixed with 1 N Folin-Ciocalteu reagent with 1.5 mL of 20% of sodium carbonate (Na_2_CO_3_). The mixture was incubated at room temperature for 30 min and after that absorbance was measured at 765 nm in a UV–visible spectrophotometer (Electronics, India).

The content of the total flavonoid was assessed using the aluminum chloride (AlCl_3_) method. The crude fungal extract (10 mg mL^−1^) was mixed with 2 mL of distilled water and 0.15 mL of sodium nitrite (5% NaNO_3_) solution and incubated for 6 min at room temperature. After 6 min, 10% AlCl_3_ was added, and the solution was left undisturbed for 6 min at room temperature. The absorbance was measured at 510 nm in the UV–vis spectrophotometer (Electronics, India). The total phenol (mg g^−1^ of GAE) and flavonoid (mg g^−1^ of RE) content was calculated by comparing it with a standard of gallic acid and rutin, respectively.

#### Extracellular enzymatic activity

All the endophytic fungi isolates were tested for their ability to produce extracellular enzymes such as cellulase, laccase, amylase, lipase, and protease. The endophytic fungal strains were grown on PDA (HiMedia, India), pH 6.7 at 28 °C for 7 days. After preparing fresh culture, the mycelial plugs were cut into 5 mm in diameter with the help of a cork borer and individually placed on a solid media containing a specific substrate. The activity of each enzyme was detected^72^.

##### Cellulase

For the screening of cellulase production, the fungal cultures were inoculated on yeast extract peptone medium (YEPD) supplemented with 0.5% Na-carboxymethyl cellulose (CMC) and incubated at 25 ± 2 °C for 5–6 days. Following incubation, the culture plates were stained with 0.2% Congo red and destained with 1 M NaCl for 15 min. The clear zone surrounding the colony indicated the presence of cellulase activity^72^.

##### Laccase

For the laccase activity, the fungal cultures were inoculated on a glucose yeast peptone agar (GYP) medium containing 1-naphthol (0.05 g L^−1^). Post incubation at 25 ± 2 °C for 5–6 days, the plates containing 1-naphthol become oxidized and produce blue color on the growth medium, displaying laccase activity.

##### Amylase

Amylase activity was evaluated by growing the fungi on GYP media at pH 6, at 25 ± 2 °C for 5–6 days. After incubation, the plates were stained with a 1% iodine solution containing 2% potassium iodide (KI). A clear zone around the colony indicated the presence of amylase.

##### Lipase

The fungal were incubated on peptone agar medium (peptone 10.0 g; NaCl 5.0 g; CaCl_2_·H_2_O 0.1 g; agar 16 g; pH 6.0, in 1 L of distilled water) supplemented with tween 20 (1%). Lipase-producing fungi were characterized by the presence of a clear zone surrounding the colony.

##### Protease

The fungal cultures were grown on GYP media supplemented with 0.4% gelatin at pH 6.0. Further, 8% of the gelatin was sterilized separately and added to the GYP medium. After incubation at 25 ± 2 °C for 5–6 days, the plates were flooded with saturated aqueous ammonium sulphate. The development of precipitation around the fungal mycelia indicates the presence of protease.

#### Plant growth promotion seed germination percentage and seedling germination

The five selected endophytic fungi were tested for their potential in the promotion of seed germination of tomato (S-22 cultivar). The seeds were surface sterilized with 70% ethanol (v/v) for 1 min, subsequently treated with 1% (v/v) sodium hypochlorite solution (NaClO) for 3–4 min, and rinsed with sterilized deionized water (dH_2_O). Sterilized seeds were further immersed in 5 mL PDB suspension of five respective fungal isolates for 12 h., while suspension containing only PDB served as control. The following day, the seeds were kept in the dark for germination in petri plates at room temperature, and the germination percentage was screened at every 2-day interval until 13 days. The germination percentage was calculated using the following formula.

$$Seed\,\, Germination\,\, (\%)=\frac{No.\,\, of\,\, germinated\,\, seeds}{Total\,\, no.\,\, of\,\, seeds}\times 100.$$

Bio-primed tomato seeds with five endophytic fungal suspensions were evaluated for the advancement of growth and biomass. Seeds were grown in germination trays following a 14 h light/12 h dark cycle at 28 °C for 15 days and after 15 days, different plant growth-promoting characteristics were recorded such as root and shoot length, fresh weight, dry weight, and seed vigor index (SVI)^73^. SVI was calculated using the following formula:

$$SVI=Germination\,\, percentage\times\,\, (Mean\,\, shoot\,\, length +Mean\,\, root\,\, length).$$

#### Pathogenicity assay

Healthy fruits of chilli (*Capsicum annum*), strawberry (*Fragaria* × *ananassa*) and tomato (*Solanum lycopersicum*) were procured from local vegetable and fruit markets. The endophytic fungi, *C. yulongense* Ai1, *C. cobbittiense* Ai2*, C. alienum* Ai2.1, *C. cobbittiense* Ai3, and *F. equiseti* Ai4, were evaluated for their pathogenic ability on their respective hosts. Out of the five isolates, four endophytic fungi belonging to genera *Colletotrichum* spp. pathogenicity assay was carried out on chilly and strawberry. Concurrently, pathogenicity of *F. equiseti* Ai4 was carried out on tomato fruit and leaves.

Spore suspension of all the endophytic fungi isolates were prepared by growing the fungus in PDA plates for seven days. Conidial suspension was made in distilled water and spore density was adjusted to (1 × 10^6^) conidia mL^−1^ before use with the help of haemocytometer. All fruits and leaf samples were surface sterilized with 70% (v/v) ethanol for 1 min followed by 1% (v/v) sodium hypochlorite (NaClO) for 4 min and then washed three to four times with sterile deionized water (dH_2_O)^74^. Excess waters were soaked through sterile blotting sheets followed by air drying under laminar air flow for half an hour. Finally, the fruits and leaf were wounded with a sterile needle, and the conidial suspension of the respective fungi was inoculated on the wounded region drop wise. The fruits and leaf samples were incubated in a moistened box for ten days and the appearance of disease symptoms were recorded. The wounded fruits sprayed with sterile water served as a control. The incubation was carried out at room temperature in dark conditions and leaves were sprayed with sterile dH_2_O at different time intervals to maintain humidity.

#### Data analysis

Significant differences were calculated by comparing mean differences and one-way ANOVA was carried out using SPSS 16. In addition, principal component analysis (PCA) was used to examine the distribution of different isolates based on different biochemical tests such as secondary metabolite production, plant growth promotion activity, and extracellular enzyme production. The biplot was prepared by using the XLSTAT by Addinsoft 2022 software to investigate the degree of similarity to the different endophytic fungal strains.

## Results

### Isolation and molecular identification of endophytic fungi

**Figure 2:**
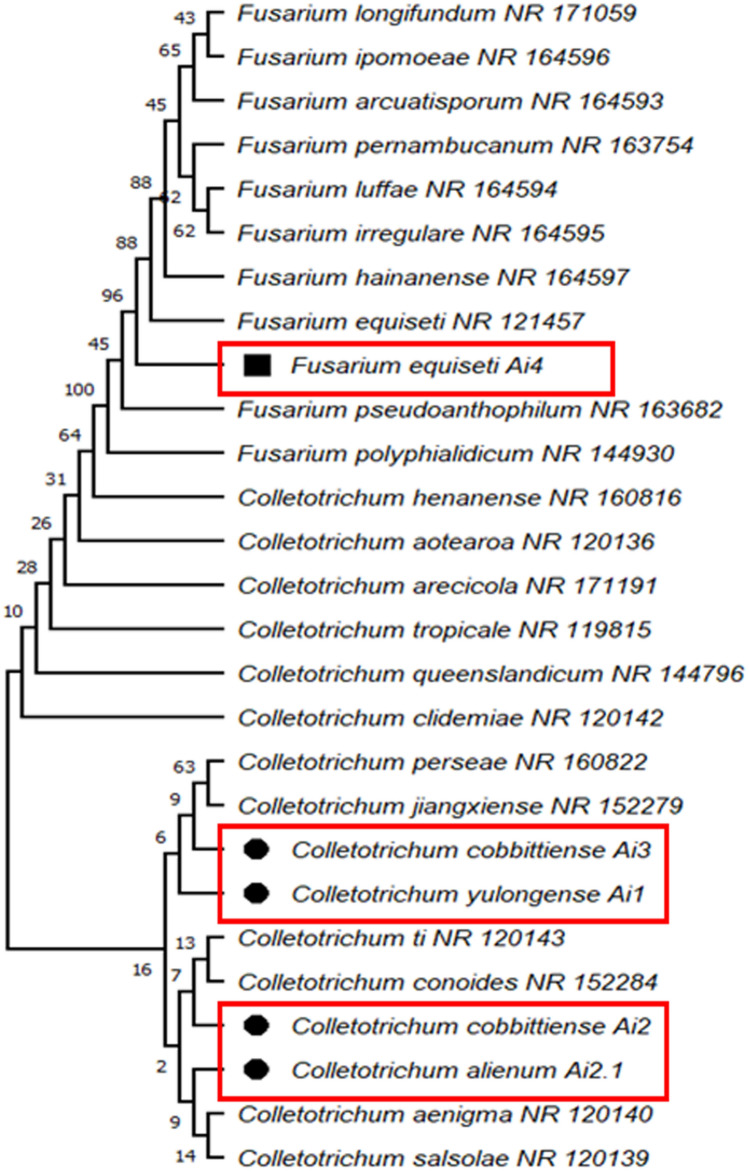
Phylogenetic relationship of isolated endophytic fungal strains with reference sequences retrieved from NCBI gene bank database. The tree was constructed using MEGA X with the neighbour-joining method and bootstraps values (1000 replicates). The dendrogram depicts the relative position of five isolates and their closet relative (highlighted in the red).

A total of ten endophytic fungi were isolated from the mature leaves of the *A. indica* plant. Five endophytic fungal isolates were selected on the basis of morphological and cultural differences (Fig. 1iv) for molecular identification using ITS rDNA (ITS1 and ITS4) sequence analysis. The five fungal endophytes were identified as *Colletotrichum yulongense* Ai1 (ON077594), *Colletotrichum cobbittiense* Ai2 (ON077429), *Colletotrichum alienum* Ai2.1 (ON077351), *Colletotrichum cobbittiense* Ai3 (ON063344), and *Fusarium equiseti* Ai4 (ON063345) (Table 1). All the sequences of identified fungal isolates were submitted to the NCBI Genbank database. The isolates have a sequence similarity of ≥ 98%, which was confirmed by BLAST analysis in the NCBI database (Table 1). Phylogenetic analysis of the selected endophytic fungal strains was performed using 1000 replicates of bootstrap analysis according to the neighbor-joining method to evaluate the relativity of the branches of the created tree. The sequences obtained from the isolates were compared with reference fungal taxa from the NCBI database. All consensus sequences were aligned and the phylogenetic tree of the isolates was constructed (Fig. 2). The results showed that all five isolates belonged to the genus *Colletotrichum* (99%) and the other was identified as *Fusarium* (1%) (Fig. 2).

**Table 1:** Endophytic fungal isolates identified based on ITS sequences.

Isolates	NCBI GenBank accession number	Sequence identity percentage (%)	Homolog sequences
Ai1	ON077594	98.81	*Colletotrichum yulongense*
Ai2	ON077429	99.47	*Colletotrichum cobbittiense*
Ai2.1	ON077351	100	*Colletotrichum alienum*
Ai3	ON063344	100	*Colletotrichum cobbittiense*
Ai4	ON063345	99.58	*Fusarium equiseti*

### Plant growth promoting traits

#### Phosphate solubilization and NH_3_ production

**Figure 3:**
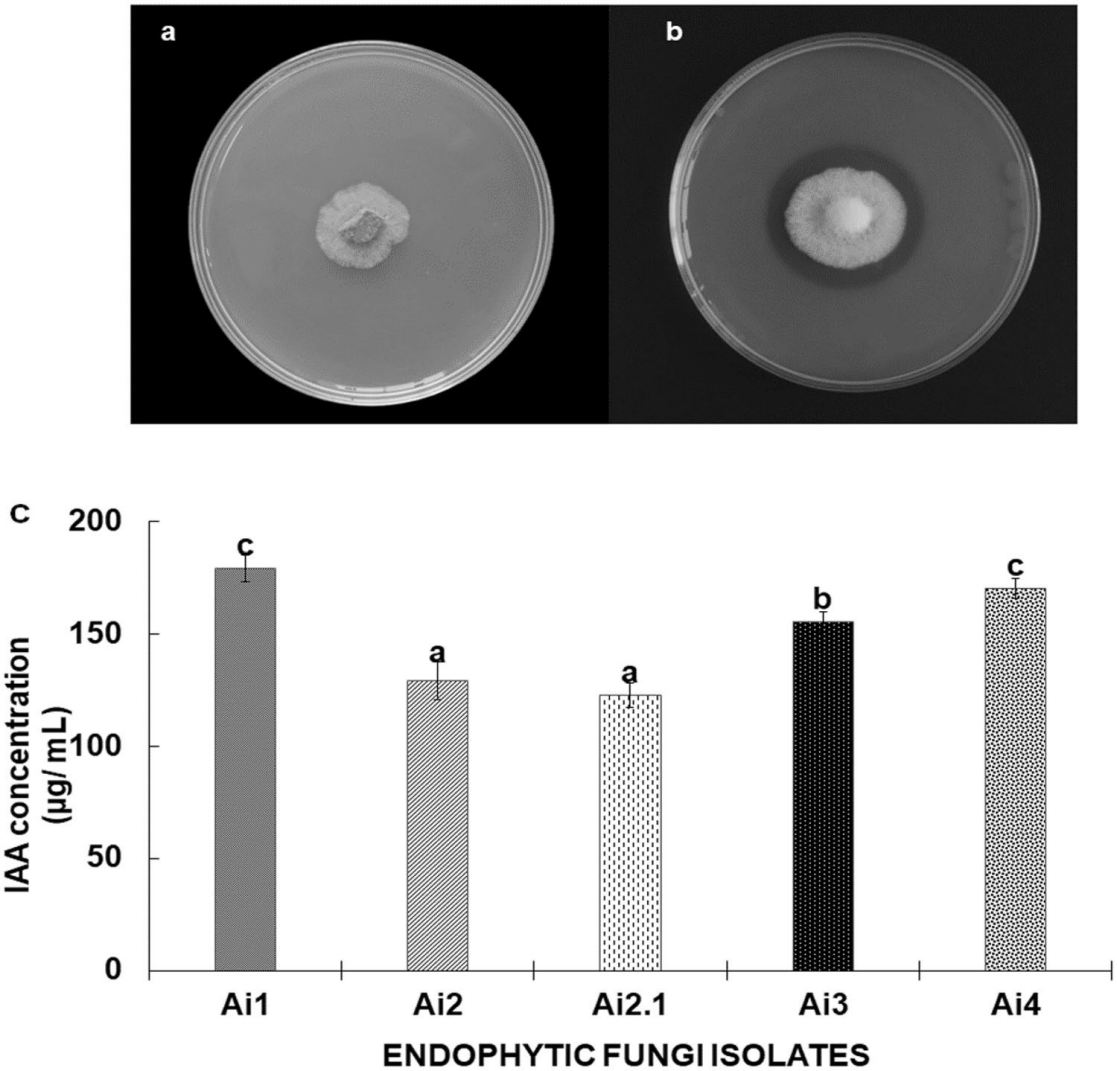
Endophytic fungal isolates showing phosphate solubilisation on Pikovskaya’s agar media plates incubated at 25 ± 2 °C (**a**) No P-solubilisation; (**b**) P-solubilisation as indicated by presence of clear zone around fungal stub and (**c**) quantitative analysis of IAA production by different endophytic fungal isolates. Values are the mean from three replicates ± SD. Different lowercase letters denote significant differences at P ≤ 0.05 analyzed by the Duncan test in SPSS 16.

Significant differences in phosphate solubilizing activity were noticed by growing the endophytic fungal isolates on Pikovskaya agar media containing Ca_3_ (PO_4_) as an inorganic phosphate source (Fig. 3a,b). It was observed that out of the five isolates, only the four isolates *C. yulongense* Ai1, *C. cobbittiense* Ai2, *C. cobbittiense* Ai3, and *F. equiseti* Ai4 were able to form clear zones, suggesting considerable phosphate solubilization activity. The isolate *C. cobbittiense* Ai2 recorded the maximum solubilization and showed a clear zone of 39 mm, followed by *C. yulongense* Ai1 (34 mm), *C. cobbittiense* Ai3 (31 mm), and *F. equiseti* Ai4 (24 mm) (Table 2).

**Table 2:** Recognition of plant growth promoting traits of different endophytic fungal isolates. Values are the mean ± SD (n = 3) Different letters (a, b, c, and d) denote that mean values are significantly different (p ≤ 0.05) by the Duncan test. +++, deep yellow to brownish color (i.e., maximum ammonia production).

Isolates	Phosphate solubilization activity (clear zone diameter in mm)	NH_3_ production
Control	0^a^	0
Ai1	34.00 ± 1.00^d^	+++
Ai2	39.00 ± 1.00^e^	+++
Ai2.1	0	+++
Ai3	31.00 ± 1.50^c^	+++
Ai4	24.00 ± 1.00^b^	+++

Endophytic fungal isolates were also evaluated for NH_3_ production on the basis of their ability to change color of the inoculation growth medium with addition of Nessler’s reagent. The fungal isolates *C. yulongense* Ai1, *C. cobbittiense* Ai2, *C. cobbittiense* Ai3, and *F. equiseti* Ai4 showed considerably high efficiency in ammonia production under in vitro conditions (Table 2).

#### IAA production

The present study showed noticeable IAA production by the endophytic fungal isolates *C. yulongense* Ai1, *F. equiseti* Ai4, *C. cobbittiense* Ai3, *C. cobbittiense* Ai2, and *C. alienum* Ai2.1 (Fig. 3c). The isolate *C. yulongense* Ai1 isolates (179.22 µg mL^−1^) showed the highest amount of IAA followed by *F. equiseti* Ai4 (170.33 µg mL^−1^), *C. cobbittiense* Ai3 (155.33 µg mL^−1^), *C. cobbittiense* Ai2 (129.22 µg mL^−1^), and *C. alienum* Ai2.1 (122.55 µg mL^−1^) in the liquid medium supplemented with L-tryptophan when shaken in the dark at 25 ± 2 °C. It has been observed from the results that the fungal isolates produced significantly more IAA in the presence of the precursor molecule tryptophan in the growth media.

#### Screening for the secondary metabolites

The study showed the presence of different secondary metabolites in fungal extracts of various isolates (Table 3). The presence of alkaloids, flavonoids, phenolics, terpenoids, and steroids was observed in the fungal extract synthesized from *C. yulongense* Ai1, *C. cobbittiense* Ai2, and *C. alienum* Ai2.1. In contrast, the fungal extract from isolate *C. cobbittiense* Ai3 screened positive for flavonoids, phenolics, and terpenoids while negative for steroid production. Additionally, the fungal extract from isolate *F. equiseti* Ai4 demonstrated positive for alkaloids, flavonoids, terpenoids, and steroids but tested negative for phenolic production (Table 3).

**Table 3:** Screening for the detection of different secondary metabolites in the crude extracts of endophytic fungal isolates of *A. indica*.

Isolates	Alkaloids	Flavonoids	Phenolics	Terpenoids	Steroids	Saponins
Ai1	+	+	+	+	+	−
Ai2	+	+	+	+	+	−
Ai2.1	+	+	+	+	+	−
Ai3	+	+	+	+	−	−
Ai4	+	+	−	+	+	−

‘+’, indicate positive result; ‘−’, indicate negative result.

#### Quantitative estimation for total phenol and flavonoids content

The quantitative investigation revealed significant differences in the total phenolic and flavonoid contents of the different extracts (Table 4). The highest total phenolic concentration was observed in the fungal extract of *C. cobbittiense* Ai2 (25.98 mg g^−1^) followed by *F. equiseti* Ai4 (25.80 mg g^−1^), *C. alienum* Ai2.1 (21.93 mg g^−1^), *C. cobbittiense* Ai3 (21.30 mg g^−1^), and *Colletotrichum yulongense* Ai1 (20.10 mg g^−1^). On the other hand, maximum total flavonoid concentration was observed in extracts of *F. equiseti* Ai4 (17.86 mg g^−1^) followed by *C. cobbittiense* Ai2 (14.58 mg g^−1^), *Colletotrichum yulongense* Ai1 (12.47 mg g^−1^), *C. cobbittiense* Ai3 (3.81 mg g^−1^) and *C. alienum* Ai2.1 (3.71 mg g^−1^) (Table 4).

**Table 4:** Quantitative estimation of total phenolic and flavonoid content in the crude extracts of different endophytic fungal isolates.

Isolates	Total phenolic content (mg g^−1^ of GAE)	Total flavonoids content (mg g^−1^ of GAE)
Ai1	20.10 ± 0.01^a^	12.47 ± 0.00^b^
Ai2	25.98 ± 0.05^a^	14.58 ± 0.00^c^
Ai2.1	21.93 ± 0.01^b^	7.09 ± 0.06^a^
Ai3	21.30 ± 0.01^a^	12.36 ± 0.06^a^
Ai4	25.80 ± 0.01^b^	17.86 ± 0.00^d^

Values are the mean from three replicates ± SD. Different letters (a, b, c, and d) denote that mean values are significantly different (p ≤ 0.05) by the Duncan test.

#### Extracellular enzymatic activity

**Figure 4:**
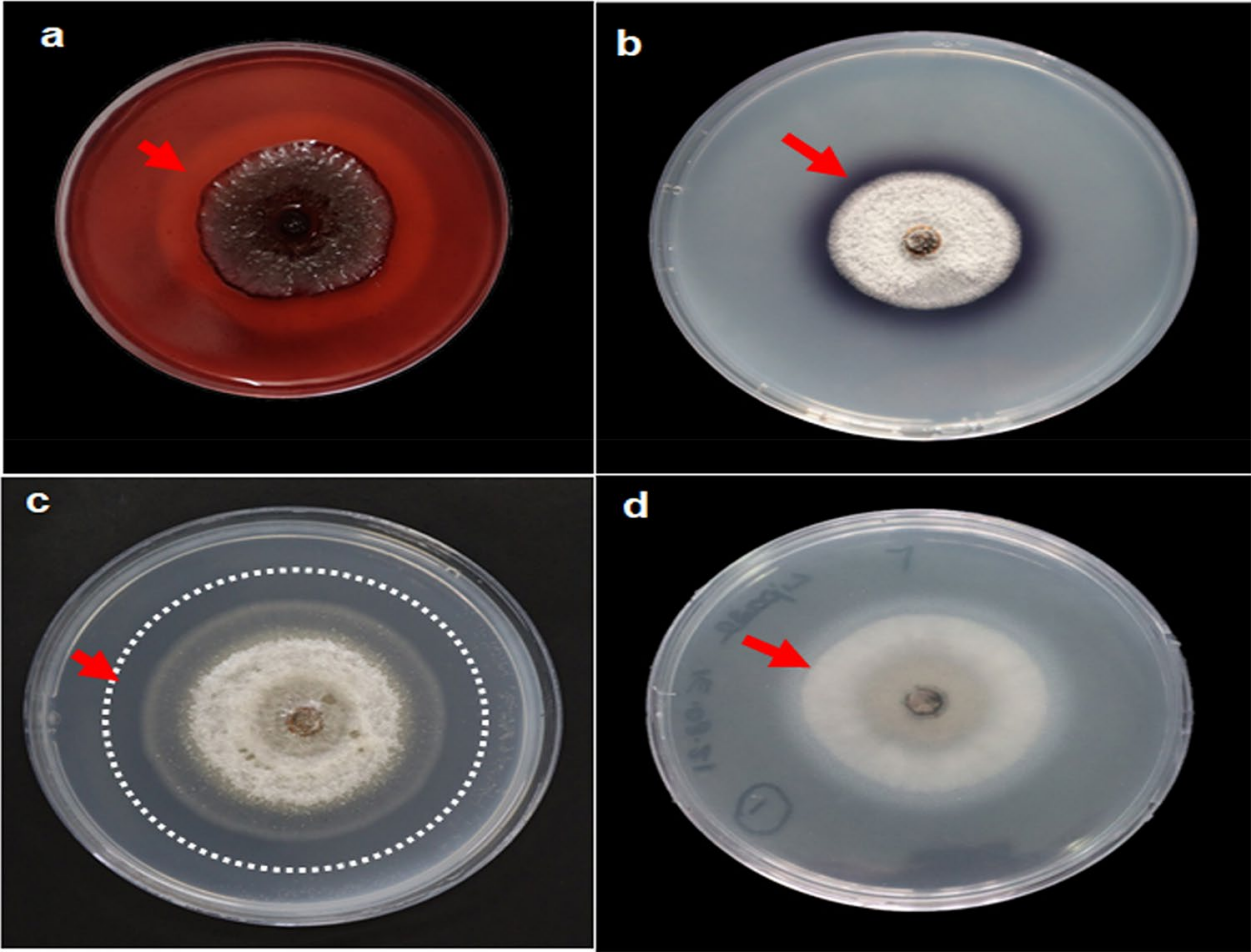
Representative picture of endophytic fungal isolate with positive results for the presence of extracellular enzymatic activity (**a**) cellulases; (**b**) laccase; (**c**) pectinase; and (**d**) lipase. Production of enzymes in the media plates have been marked by the presence of dotted circles and arrows.

All the fungal isolates were examined and screened for their extracellular enzyme synthesis, including amylase, protease, lipase, cellulase, and pectinase (Fig. 4). The data showed that the four endophytic isolates *C. yulongense* Ai1, *C. cobbittiense* Ai2, *C. alienum* Ai2.1, and *C. cobbittiense* Ai3 exhibited considerable production of cellulases (Table 5). Laccase activity was shown by *Colletotrichum yulongense* Ai1 and *F. equiseti* Ai4 while all isolates (*C. yulongense* Ai1, *C. cobbittiense* Ai2, *C. alienum* Ai2.1, *C. cobbittiense* Ai3, and *F. equiseti* Ai4) tested negative for amylase production (Table 5). None of the isolates tested positive for amylase activity, indicating that enzyme production varies among fungal species depending on host habitat. In contrast, all the fungal isolates (*Colletotrichum yulongense* Ai1, *C. cobbittiense* Ai2, *C. alienum* Ai2.1, *C. cobbittiense* Ai3, and *F. equiseti* Ai4) showed positive results for the lipase production (Table 5). Nevertheless, two of the endophytic fungal isolates (*Colletotrichum yulongense* Ai1 and *C. cobbittiense* Ai3) showed positive tests for protease activity.

**Table 5:** Extracellular enzymatic activities of different endophytic fungal isolates of *A. indica*.

Isolates	Cellulase	Laccase	Amylase	Lipase	Protease
Control	−	−	−	−	−
Ai1	+	+	−	+	+
Ai2	+	−	−	+	−
Ai2.1	+	−	−	+	+
Ai3	+	−	−	+	−
Ai4	−	+	−	+	−

‘+’, indicate positive result; ‘−’, indicate negative result.

#### Principal component analysis

**Figure 5:**
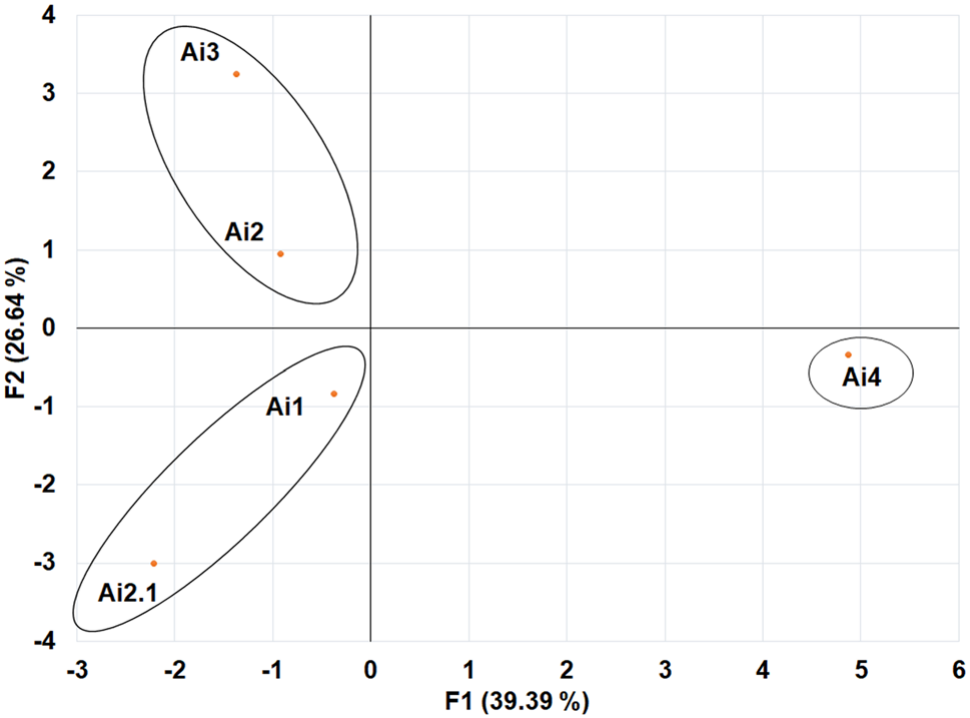
Principal component analysis (PCA) for clustering of endophytic fungal isolates based on various biochemical characteristics. Five isolates were clustered into three groups based on their biochemical properties.

PCA analysis revealed that the five isolates were separated into three clusters based on different biochemical characteristics (Fig. 5). Two major cluster includes *Colletotrichum* (Cluster I and II), while cluster III bear *Fusarium* genera. Within cluster I, *C. cobbittiense* Ai3 is distantly located from the centroid in comparison to *C. alienum* Ai2 which is close to the centroid. Similarly, *Colletotrichum yulongense* Ai1 in cluster II is close to the centroid while Ai 2.1 is distantly located from the centroid. Furthermore, the isolate *F. equiseti* Ai4 which formed an independent cluster is also located distantly from centroid (Fig. 5).

### Plant growth promotion

#### Seed germination percentage and seedling germination

**Figure 6:**
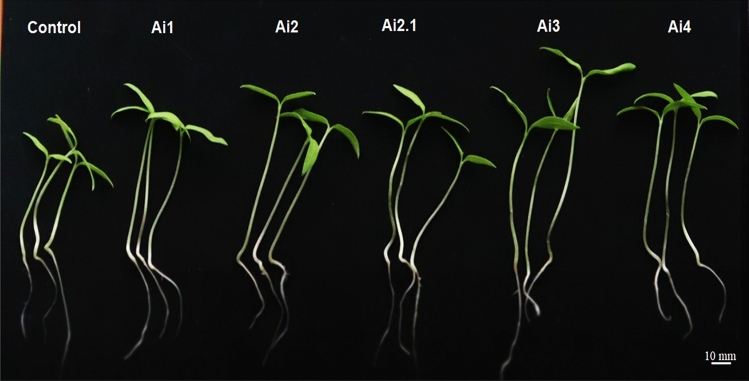
Effect of seed biopriming with selected fungal isolates on the shoot and root growth of tomato seedlings.

Biopriming of tomato seeds with endophytic fungal isolates led to considerably higher germination rate than the control (without bioprimed seeds) (Table 6). Seeds bio-primed with the endophytic isolate *C. cobbittiense* Ai2 showed the highest germination percentage than control (Table 6). Likewise, all the bioprimed tomato seeds showed a significant increase in shoot and root growth as compared to control (Fig. 6). Among the isolates the highest root length was observed when tomato seeds treated with isolate *C. cobbittiense* Ai3 followed by *F. equiseti* Ai4 (3.16 cm) and *C. alienum* Ai2.1 (3.07 cm). The maximum increase in the shoot length was observed in the seedlings treated with *C. cobbittiense* Ai3 (4.23 cm) in comparison to control seedlings. Highest fresh and dry weights were observed in seedlings bioprimed with *C. cobbittiense* Ai2 in comparison to non-primed seedlings (Table 6). The seed vigor index of the seeds also increased in inoculated seedlings as compared to control grown tomato seedlings (Table 6). The highest seed vigor index of 555.35 in *C. cobbittiense* Ai3 bio-primed tomato seedlings, which was significantly higher than seedlings bio-primed with other isolates.

**Table 6:** Effect of different isolates on modulation of germination, growth and development of tomato seedlings through bio-priming. Values are the mean ± SD (n = 3) Different lowercase letters (a, b, c, and d) denote that mean values are significantly different (p ≤ 0.05) by the Duncan test.

Isolates	Seedling germination (%)	Root length (cm)	Shoot length (cm)	Seed vigour index (SVI)	Fresh weight (gm)	Dry weight (gm)
Control	50.0	2.93 ± 0.43^a^	3.31 ± 0.41^a^	312.00 ± 13.31^a^	0.177 ± 0.028^a^	0.008 ± 0.002^a^
Ai1	73.7	2.91 ± 0.47^a^	3.69 ± 0.39^b^	486.78 ± 31.98^b^	0.189 ± 0.007^ab^	0.008 ± 0.001^a^
Ai2	80.0	3.03 ± 0.41^a^	4.14 ± 0.38^cd^	574.00 ± 31.22^c^	0.227 ± 0.017^c^	0.011 ± 0.001^b^
Ai2.1	73.1	3.07 ± 0.40^a^	3.93 ± 0.33^bc^	512.43 ± 22.97^b^	0.202 ± 0.009^bc^	0.010 ± 0.002^ab^
Ai3	72.5	3.42 ± 0.38^b^	4.23 ± 0.47^d^	555.35 ± 18.11^c^	0.217 ± 0.018^c^	0.010 ± 0.002^ab^
Ai4	70.0	3.16 ± 0.51^ab^	4.18 ± 0.50^cd^	514.50 ± 16.42^b^	0.211 ± 0.022^bc^	0.009 ± 0.001^ab^

#### Pathogenicity assay

**Figure 7:**
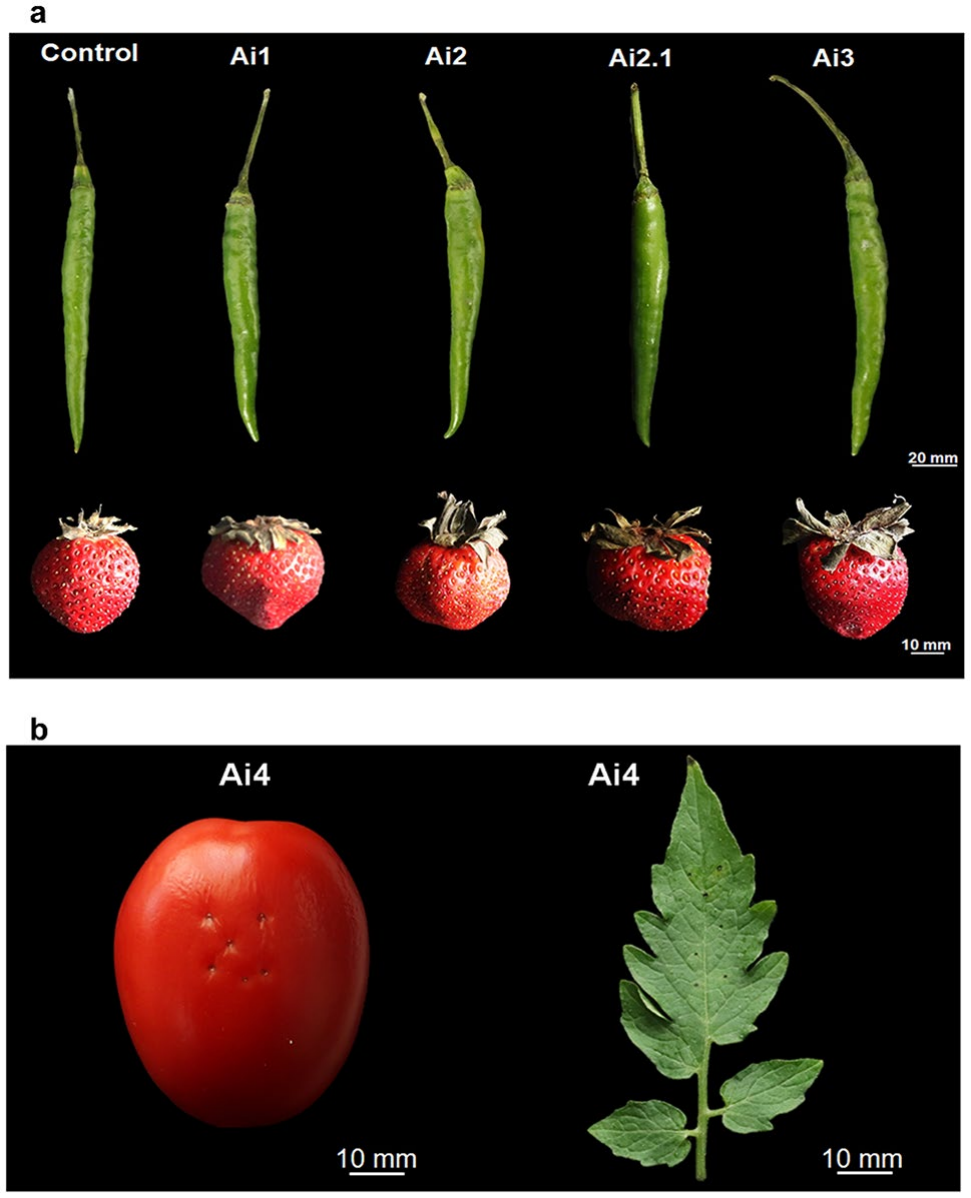
Pathogenic assay of selected fungal isolates on (**a**) fruits of chilli and strawberry (**b**) leaf and fruit of tomato. No lesions were observed after 10 days post inoculation (dpi) in any of the fruits confirming non-pathogenic behaviour.

Non-pathogenic effects of endophytic fungal isolates *Colletotrichum* spp. (*C. yulongense* Ai1, *C. cobbittiense* Ai2*, C. alienum* Ai2.1, *C. cobbittiense* Ai3) was observed on fruits of chilly and strawberry fruits as evident due to absence of lesions even after ten dpi (Fig. 7a). Correspondingly, no lesion was visible on leaf and fruit of tomato plants inoculated with the fungal isolate *F. equiseti* Ai4 thus confirming non-pathogenic effect (Fig. 7b).

#### Discussion

A total of ten endophytic fungi were isolated from the leaves of *A. indica* and five among them showed significant characteristics in terms of in-vitro plant growth promotion, secondary metabolite production, extracellular enzyme production and anti-bacterial activity. These five isolates were tested positive for in-vivo seed germination and growth of tomato. On the basis of ITS sequencing, the five isolates were identified as *Colletotrichum yulongense, C. cobbittiense, C. alienum, C. cobbittiense and Fusarium equiseti* and phylogenetically analyzed for their closeness. The present work stated for the presence of major foliar endophytic fungi mainly belonging to genera *Colletotrichum* and *Fusarium* sp. A number of previous studies have reported the presence of these two fungal genera as a dominant group of endophytes residing in association with different medicinal plants^75–78^. These two genera offered many benefits to plants when live as an endophyte and assist medicinal plants in imparting various characteristics as seen in the present study. Wu et al.^79^, isolated 970 endophytic fungi from the medicinal plant *Litsea cubeba* and studied their diversity as well as assessed their anti-microbial potential. Interestingly, the work showed *Colletotrichum* as the most dominant endophytic fungi with substantial anti-microbial activity. Similarly, *Colletotrichum* sp. BS4 has been shown to be one of the dominant endophytic fungi associated with Chinese medicinal plant *Buxus sinica*^80^. From the present study, it has been observed from PCA analysis that *Colletotrichum* isolates are grouped together in two different clusters although they are phylogenetic distant. Sharma et al.^81^ also revealed the occurrence of *C. gloeosporioides* as a dominant endophytic fungus isolated from *Syzygium cumini* plants. These *C. gloeosporioides* have been shown to produce bioactive metabolites and extraction was done from the fungal extract. In a different study, twenty-two distinct species of *Colletotrichum* strains (*C. boninense*, *C. orchidophilum, C. jiangxiense*, *C. fructicola* and *C. citricola*) have been isolated from orchid *Dendrobium cariniferum, D. catenatum* and *D. harveyanum*^82^. Recently, Nazir et al.^83^, reported *Fusarium* sp. as a dominant endophytic fungus isolated from *Olea europaea*. Endophytic fungus *Fusarium oxysporum* was isolated from the leaves of *Otoba gracilipes*^84^. Similarly, Lu et al.^85^, demonstrated *Fusarium* sp. HJT-P-2 as dominant endophytic fungi studied from *Rhodiola angusta.* Interestingly, Xiao et al.^86^, discovered new secondary metabolites such as pyrone derivative (1–5) from the dominant endophytic fungi *Fusarium* sp. HP-2 isolated from Agarwood Qi-Nam plant. *Fusarium* sp. has been separately grouped into different clusters as seen from the PCA biplot that provides a clear indication of the uniqueness of this isolate in various biochemical characteristics. The present study also demonstrated *Colletotrichum* and *Fusarium* genera as an important endophytic fungal strain with significant potential to be utilized as biofertilizers along with the industrial value.

Bioprospecting of these fungal endophytes associated with medicinal plants will have momentous applications in pharmacology due to the presence of secondary metabolites in their crude fungal extracts. In the present study, presence of alkaloids, flavonoids, phenolics, terpenoids, and steroids were observed in crude fungal extracts of all the five isolates Mahmud et al.^87^ reported the presence of bioactive metabolites such as flavonoids, anthraquinones, coumarins, isocoumarins, etc. in endophytic fungal extracts of *C. gloeosporioides, Fusarium solani, C. tropicale,* and *C. siamense*. Similar results were shown by Singh et al.^88^, that demonstrated the presence of phenolics, and terpenoids in the crude fungal extracts of isolated endophytic fungus *Fusarium* sp. from the folk medicinal plant *Cissus quadrangularis*. Comparable reports were observed from the work of Devi et al.^89^, that showed presence of alkaloids, phenols, flavonoids, tannin, and glycosides from crude fungal extracts of isolated endophytic fungi *Penicillium* sp. from medicinal plant *Centella asiatica*. Ladoh-Yemeda et al.^90^, also reported presence of flavonoids, anthraquinones, tannins, phenols, steroids, coumarins, and terpenoids in crude fungal extracts of the *Phragmanthera capitata.* Furthermore, Gautam et al.^91^, showed positive result for total phenol and flavonoid concentration in crude fungal extracts of *Nigrospora sphaerica* (EHL2), isolated from the leaf tissues of *Euphorbia hirta*. Our results are in corroboration with various previous studies that showed the presence of alkaloids, flavonoids, phenols, terpenoids, and sterols in different fungal extracts^87,92–94^.

Apart from their role in pharmacology, a number of fungal endophytes play a huge role in industrial microbiology for the commercial production of enzymes that are being used in various industries^48,49,95^. From the present work, it can well establish that *Colletotrichum* and *Fusarium* resulted in extracellular enzyme production such as cellulases, laccases, amylases, lipases, and proteases under qualitative screening. Enzyme production by endophytic fungal isolates was reported by Toghueo et al.^72^, in which 21 genera of endophytic fungi (including *Fusarium* and *Colletotrichum*) isolated from three medicinal plants such as *Cananga odorata*, *Terminalia catappa*, and *T. mantaly* showed positive tests for amylase, cellulase, lipase, and laccase enzymatic activity. In a different study, it was observed that the endophytic fungi *Penicillium* sp. L3 and *Colletotrichum* sp. S1 isolated from *Euterpe precatoria* exhibited significant production of hydrolases enzymes such as amylases, lipases, cellulases, and pectinases^96^. All these hydrolysing enzymes like cellulases have been widely used in agricultural waste, paper pulp, biofuel, detergent industry, fermentation, and other industries^97–102^. Similarly, microbial amylases have been used for commercial-industrial applications like starch modification, textile industrial processing, fermentation, pre-treatment, etc.^103^. Laccases are well known for their role in biodegradation and isolation of lacasses from endophytic fungal isolates can provide a huge boon to industrial biotechnology^104,105^ demonstrated optimization and extraction of laccase enzyme from an endophytic fungus *Irpex lacteus* isolated from *Euphorbia milii.*

One of the significant characteristics shown by these five endophytic fungal isolates is plant growth promotion as seen by their ability to stimulate IAA production, NH_3_ production, and facilitated phosphate solubilization under in vitro. Plant roots in most cases are unable to solubilize insoluble inorganic forms of phosphate in the soil; therefore, association with endophytic fungi provides efficacy to host plant for conversion of phosphorus from an unavailable form to an available form^106^. A similar work by Khalil et al.^41^, demonstrated P- solubilisation by three endophytic fungal isolates *Penicillium crustosum* EP-2, *Penicillium chrysogenum* EP-3, and *Aspergillus flavus* EP-14. Chand et al.^107^, revealed the effectiveness of different endophytic fungi isolated from orchid *Vanda cristata* in P-solubilization. IAA production is considered as a major phytohormone responsible for cell and root growth, nutrient uptake, etc.^108,109^. Recently, Khan et al.^110^, isolated endophytic fungi *Acremonium* sp. from *Lilium davidii* and it was observed that the fungus has potent ability for P-solubilisation as well as IAA production. Fouda et al.^111^, reported IAA production by endophytic fungi *Penicillium chrysogenum* Pc_25 and *Alternaria alternate* Aa_27 when tryptophan was used as a precursor molecule. Galeano et al.^112^, isolated *Aspergillus niger* 9-P from native forage grass, and these strains were capable of solubilizing phosphates and successful in the production of ammonia and IAA. Fungal endophytes belonging to the genera *Neopestalotiopsis*, *Trichoderma, Fusarium, Colletotrichum, Myrothecium, Chaetomium, Alternaria, Phoma, Curvularia, Cladosporium, Neodidymelliopsis,* and *Aspergillus* isolated from the medicinal plants *Jasminum sambac, Camellia sinensis,* and *Ocimum basilicum* have shown significant plant growth-promoting activities by producing IAA, 1-aminocyclopropane-1-carboxylic acid (ACC) deaminase enzyme, and siderophores^113^. Sharma et al.^114^ isolated endophytic fungi *Clonostachys pseudochroleucha* (ARW2), *Parathyridaria percutanea* (N4), *Curvularia lunata* (H1B), *F*. *proliferatum* (H22) and *F. equiseti* (G8) from *Dioscorea bulbifera* and displayed their positive role in plant growth promoting traits such as siderophore production, phosphate solubilization, and HCN production respectively. In a different study, endophytic fungi isolated from the healthy leaves of *Biserrula pelecinus*, and *Ornithopus compressus* exhibited plant growth-promoting traits under in vitro as well in vivo^115^. Simultaneously, endophytic fungi *Colletotrichum gloeosporioides* (DJL-6), *Trichoderma tomentosum* (DJL-9), *Colletotrichum godetiae* (DJL-10) and *Talaromyces amestolkiae* (DJL-15) isolated from the tubers of *Cremastra appendiculata* demonstrated significant production of IAA, β-1,3-glucanase, siderophore. The isolated endophytic fungi strong nitrogen fixation ability and potassium dissolution which augment the growth of *C. appendiculata* and soybean seedlings^116^. A study revealed that endophyte fungus *Penicillium citrinum* isolated from the leaves of wheat variety PBW 725 promoted the growth attributes and improved the adaptability under stress by the production of IAA, gibberellic acid, NH_4_, solubilized phosphate and zinc as well as increased production of siderophores^117^.

It has been observed that these fungal endophytes *Colletotrichum* and *Fusarium* have a vast potential to be utilized as biostimulants that endophytic fungi for enhancing plant growth and sustaining better plant health. In vitro results have been confirmed by analysing their potential in the present work through seed biopriming of tomato with five selected endophytic fungal isolates under in vivo. The present work outline the increase in percent seed germination of tomato seeds after biopriming. Concomitantly, there was significant increase in seedling growth in terms of higher root and shoot length, fresh and dry weight etc. The results are in line with previous work of various other researchers that showed similar increase in growth and development of plants through bioinoculation with fungal endophytes as well commercially used crops^118–121^. Likewise, the long-term symbiotic relationship and multiplication of endophytic fungi *Clonostachys rosea* ST1140 in solanaceous fruit vegetable pepper, tomato, and eggplant showed a 10% higher germination rate and faster growth rate as compared to the non-inoculated plants^112^. In a recent study Hatamzadeh et al.^122^, ninety-seven endophytic fungi mainly belong to genera belonging to the genus *Fusarium* and *Alternaria* were isolated from five medicinal plants namely *Anthemis altissima, Matricaria parthenium, Cichorium intybus, Achillea millefolium*, and *A. filipendulina* and most of the endophytic fungi. The study further showed that the fungal endophyte *Alternaria* (BR41) promoted higher germination and better plant growth of *Zea mays* (cv. ZP684)^122^. Zhang et al.^123^ reported enhanced number of roots, shoots, and leaves in tissue culture plantlets of ginger with application of fungal endophyte *Sarocladium strictum* GR-2. In a different study by Zhang et al.^97^, investigated the growth enhancement of the *Vaccinium macrocarpon* (cranberry) by one of its fungal endosymbionts *Codinaeella* sp. EC4 and found substantial enhancement of cranberry seedlings. The results were further correlated with the genetic expression of an intracellular fungal endophyte EC4 and it was established that the genome of fungus contains 17,582 potential protein-coding genes, of which almost 500 genes are related to plant growth^124^. There was increase in seedling height, stem and diameter along with increased shoot dry weight, total biomass, root activity and total chlorophyll of blueberry seedlings inoculated with dark septate endophytic fungi *Cladosporium cladosporioides*^125^. Furthermore, seed biopriming with endophytic fungal strain have also shown to higher seed germination rate as well as increased tolerance to salt stress^126^.

Fungal endophytes have always been a topic of research enigma to scientists as most of these fungal endophytic partners in normal case are known to be plant pathogenic like *Fusarium*, *Colletotrichum*, etc. Endophytic fungi have a symbiotic relationship with their host plants and exert non-causal pathogenic effects on their host plants by enhancing and accelerating plant growth by enhancing nutrient uptake, production of various phytohormones, protecting the plant from stresses, etc.^127^. The mechanisms by which these “nonpathogenic” microbes engage with plants are poorly understood, particularly during long-term, steady-state interactions that are more representative of plant-microbiota interactions in nature^128^. In the current study two important genera *Fusarium* and *Colletotrichum* that are normally known for causing severe plant diseases non-pathogenic as evident from the pathogenic assay on their respective known hosts chilli, strawberry and tomato fruits. Interestingly, there are previous reports that stated anti-fungal effects of endophytic fungi as seen by the work of Abdel-Motaal et al.^129^, in which four endophytic fungi *Fusarium solani*-F4-1007, *Penicillium verrucosum*-F2-1006, and *Aspergillus terreus*-F5-1008, inhibited the growth of the pathogenic *Cochliobolus spicifer* by producing anti-fungal VOCs 3,4-dihydro-2h-1,5-(3″-t-butyl) benzodioxepine, 4-(2-hydroxyethyl) phenol, and phenylethyl alcohol. In another work in vitro pathogenicity testing of endophytic fungi revealed that *Trichoderma phayaoense* has the potential as a biocontrol agent for gummy stem blight and wilt of *Cucumis* melo (muskmelon) and could effectively increase fruit weight, diameter, circumference, and total soluble solid without affecting fruit quality parameters.

In conclusion, the present study laid the foundation for the identification and screening of many endophytic fungal strains from *A. indica* plants for multiple roles in bioactive secondary metabolites production, plant growth promotion, and extracellular enzyme production. These fungal endophytes will provide an eco-friendly approach for sustaining the growth and development of crop plants as biostimulants in the present climate change scenario. The isolates i.e., *Colletotrichum* and *Fusarium* can be utilized at a large scale for industrial and microbial biotechnology. Future studies aim to identify novel secondary bioactive compounds from these endophytic strains and decipher the molecular pathway for an increase in these secondary metabolites.

## Data Availability

The sequences of the endophytic fungi isolates have been submitted in the NCBI GenBank databases with following accession numbers ON077594, ON077429, ON077351, ON063344, ON063345 generated during the present study.
